# Time point and scale measurement of carbon sink trading market risk based on catastrophe entropy and potential function

**DOI:** 10.1007/s11356-023-31154-8

**Published:** 2023-11-25

**Authors:** Xing Yang, Zhihua Yang, Junlong Mi, Jiawen Li

**Affiliations:** 1 College of Economics of Jinan University, Guangzhou, 510630 China; 2 Economic School of Guangzhou City University of Technology, Guangzhou, 510800 China; 3 Research Base of Carbon Neutral Finance for Guangdong-Hong Kong-Macao Greater Bay Area, Guangzhou, 510800 China

**Keywords:** Carbon trading market, The principle of total entropy change, Potential function model, Risk time point, Risk scale

## Abstract

According to the principle of total entropy change of dissipative structure, the carbon trading market is defined as a nonlinear complex system that follows the law of entropy increase in this paper. Based on the potential function of sudden change theory, this paper studies the risk point and scale of the carbon trading market. The results show that (1) the theory of dissipative structure and catastrophe theory can be used as the theoretical basis of carbon financial market risk research, and its core technology can be used to measure and predict risks. (2) The risk mutation point measurement model based on the total entropy change principle and potential function technology effectively detected 16 major risk mutation points in the financial crisis, the European debt crisis, and the European new energy efficiency plan. The empirical test shows that the model has a good ability to capture abrupt changes and prediction accuracy. The fitting effect is very good. (3) The risk index value of the risk abrupt point can be calculated effectively by the risk scale measurement technique based on information entropy and the potential function surface equation. Furthermore, we judge the degree and grade of risk. From 2008 to 2021, amongst the 16 risk mutation points in the EU carbon trading market, there are three extremely high risk mutation points, seven high-risk mutation points, two medium-risk mutation points, two low-risk mutation points, and two very low risk mutation points. High risk or above grade accounted for 62.5%. Empirical analysis supports this conclusion.

## Introduction and literature review

The abnormal fluctuation and abrupt behaviour of the financial asset return rate has always been the focus of the financial field. As a quasifinancial market, the EU Carbon Emission Trading System has witnessed an increasing market volume and increasingly complex trading environment since its launch in 2005. Risk events such as quota theft, duplicate trading, and false information appeared in the market. The superposition of external shocks, such as the financial crisis, the energy crisis, war, and natural disasters, directly led to the price of the carbon trading market plummeting all the way. The carbon market price fell below 10 euros in February 2009. It fell below 5 euros in December 2011. It fell below 2 euros in 2012. It fell to 0.04 euros in April 2013. It then slowly climbed to 0.13 euros in March 2021. The violent fluctuation of carbon emission rights market prices directly impacts market operation, investor confidence, and the failure of the market supervision mechanism. This ultimately led to a complete collapse of some markets. For example, the BlueNext^1^ market permanently closed its spot and derivative trading business on December 5, 2012. It is urgent to deeply analyse the characteristics of price fluctuation in the carbon trading market, the dynamic mechanism of price fluctuation, the internal and external environmental factors causing abnormal market fluctuation and sudden behaviour, and accurately grasp the time and scale of risk occurrence.

The research on financial market risk measurement mainly goes through three development stages: (1) the traditional risk measurement stage takes variance and risk factors as the measurement index (Tian and Yang 2021; Pan 2022; Meng 2019). (2) The modern risk measurement stage represented by VaR (Fu and Zhang 2009; Zhao and Fang 2020; Müller and Righi 2022). (3) The stage of consistent risk measurement is represented by the expected shortfall (Wang and Wei 2012; Gong et al. 2019; Hoogerheide and van Dijk 2010). The application of financial physics to complex economic systems for benefit and risk analysis is the fourth stage. It started with Peters (Peters 1994), the founder of the fractal market hypothesis. He introduced fractal and chaos theory to the economy in the early 1990s and brought rescaled range analysis back into the toolbox of researchers (Cao and Li 2003; Jiang 2009). Other techniques include genetic algorithms, wavelets, and complex system theory. Wavelet and complex system analysis can be used to measure the uncertainty risk of financial assets.

The study of abnormal price fluctuations and sudden changes in the carbon market utilizing financial physics methods has just begun. For example, Pan et al. (2022) studied the jumping behaviour of carbon asset prices based on information shock. It is found that a significant information shock will lead to a large asymmetric continuous jump in carbon asset prices. Ye et al. (2021) adopted a multiscale multifractal method to study the relationship between carbon market price and economic policy uncertainty in the European Union. Anti-persistence between the two was found. Bei et al. (2021) introduced the ARJI model, which can depict time-varying dynamic characteristics and the jumping process, to investigate the time-varying jumping problem of EUA future prices. Yang and Liang (2017) explored the behavioural characteristics of the EU carbon emission rights market by using the multifractal method of multiple-scale ranges. It is found that the carbon trading market is a nonlinear dynamic system with fractal and chaotic characteristics. Lin (2016) constructed a random cusp mutation model when studying the sudden change characteristics of China’s stock market. It is proven that the mutation model is superior to the linear model and the pseudomutation logistic model in data interpretation power. Starting with the difference between classical financial theory and financial physics, Wang and Wei (2014) analysed several financial anomalies that could not be explained by classical financial theory. A new view on the study of financial physics in financial markets is proposed.

Based on the above research, this paper uses the principles and techniques of financial physics to explore the internal mechanism of system mutation behaviour. For a long time, the measurement theories of financial risk are mainly based on the classic “Portfolio Theory” (PT) of Markowits (1952) and its successors, “Capital Asset Pricing Model” (CAPM) of Sharpe (1964) et al. In terms of methods, “variance”, “mean semi-variance”, and “mean Gini” are mainly used to measure the risk. The weakness of these theories and methods is that they cannot explain the numerous “anomalies” in financial markets, such as the “weekend effect”, “intra-daily effect”, and “January effect”. So, the financial physics arises at the historic moment. The main advantage of applying financial physics to measure risk is that it can use nonlinear dynamics and complex system theory to explain and model these anomalies. Thus, it can effectively explain the operation mechanism of financial markets, asset pricing mechanism, risk measurement, and prediction.

The arrangement of this paper is as follows. The “Theoretical basis and method: entropy of dissipation theory and potential function of catastrophe theory” section introduces the research methods. It mainly includes (1) a risk mutation point measurement model based on the entropy of dissipation theory and the potential function of catastrophe theory. (2) Risk scale measurement model based on information entropy and the potential function surface equation. The “Empirical research” section uses the real-time trading data of the EU carbon trading market from 2008 to 2021 to determine and analyse the abrupt change point and the corresponding abrupt scale of the risk. It mainly includes (1) the selection of state variables and control variables and the construction of an index system. (2) The determination of internal entropy increases and external entropy flow and the embedding of potential function. (3) Measurement and analysis of the time-varying jump of risk mutation points. (4) Risk scale measurement and grade at risk time point. The “Research conclusion and enlightenment” section will discuss the correctness or misjudgement of the research conclusions.

## Theoretical basis and method: entropy of dissipation theory and potential function of catastrophe theory

Dissipative theory and catastrophe theory are both important branches of complex system theory. Entropy is the core of dissipation theory. It studies the spontaneous and autonomous evolution of a system from a disordered state to an ordered state. Catastrophe theory takes potential energy as the core. It studies the phenomena and laws of the system jumping from one stable state to another under small accidental perturbation factors.

### Total entropy change of the carbon trading system in dissipation theory

Dissipative structure theory was first proposed by Prigogine (1969). In his view, all systems in nature can be divided into closed systems and open systems. The former has no exchange of material, energy, or information with the external environment. The system is in a fixed equilibrium state. The latter is engaged in the exchange of various substances, energies, or information with the external environment. The system is in flux, forming a nonequilibrium state. This kind of open, far from equilibrium, irreversible system is a dissipative structure system. Entropy^2^ and order are two main concepts in dissipative structure theory. Entropy change is the core of dissipative structure theory. The total entropy changes of the system *ds* consist of internal entropy increase *ds*_*I*_ and external entropy flow *ds*_*E*_. When *ds* < 0, the system evolves towards a dissipative structure. The author’s previous studies have proven (Yang and Liang 2017) that the carbon sink trading market is an open and dissipative system. It can use entropy to measure the increase in system disorder caused by the carbon emission trading process. The increase in the degree of disorder of the thermal system is caused by inefficiency. In the carbon trading system, the increase in disorder is caused by the uncertainty of information in the trading process. Carbon sink trading system entropy can be understood as information entropy^3^. According to dissipative structure theory, the total entropy changes *ds* of an open system consist of internal entropy increase *ds*_*I*_ and external entropy flow *ds*_*E*_. For the carbon trading market, the total information entropy flow can also be defined as

1 $$ds=d{s}_I+d{s}_E$$

According to the principle of entropy and entropy change, the operation of any system has the characteristic of increasing entropy within the system. That is, the entropy change of the system *ds* > 0. An isolated system is an entropy-increasing system. It just goes in the direction of disordering. An open system generates entropy flow because of its constant exchange of matter and energy with the outside world. When the entropy flow value obtained from the outside is lower than the entropy increment inside the system, that is, entropy flow reduces the entropy increment inside the system, an entropy change is generated. When *ds* < 0, the system changes from disorder to order. The system structure can be judged according to the law of entropy. When *ds*_*e*_ = 0, *ds*_*i*_ = 0, the system is a static structure. When *ds*_*e*_ = 0, *ds*_*i*_ > 0, the system is a closed structure. When *ds*_*e*_ ≠ 0, *ds*_*i*_ = 0, the system is a stable structure. When *ds*_*e*_ ≠ 0, *ds*_*i*_ > 0, the system is a dissipative structure. For the increase in internal entropy *ds*_*I*_, this paper is expressed by the rate of change of carbon asset price *ds*_*r*_. It reflects the entropy increment caused by the fluctuation of the price yield of carbon quota EUA and certified emission reduction CER. Its information entropy can be expressed as $P_m=\frac1{N_m}\sum\limits_{t=1}^{N_m}\frac{\left|R_{t,m}\right|}{vol_{t,m}}$, where *P*_*m*_ is the rate of price change in month *m*, |*R*_*t*, *m*_| is the absolute rate of return on day *t* month *m*, *Vol*_*t*, *m*_ is the turnover of day *t* month *m*, and *N*_*m*_ is the nonzero transaction days in month *m*.

In this paper, the external entropy flow is measured by four indicators: macroeconomic policy *ds*_*p*_, economic development level *ds*_*l*_, energy price *ds*_*e*_, and climate change *ds*_*c*_. The formula is

2 $${\displaystyle \begin{array}{c} ds=d{s}_I+d{s}_E\\ {}=d{s}_r+d{s}_p+d{s}_l+d{s}_e+d{s}_c\end{array}}$$

*ds*_*r*_, *ds*_*p*_, *ds*_*l*_, *ds*_*e*_, and *ds*_*c*_ are measured by information entropy *e*_*j*_.

3 $$e_j=\pm k\times\sum_{i=1}^mp_{ij}\ln p_{ij}\;\left(i=1,2,\cdots,m;\;j=1,2,\cdots,n\right)$$

where $k=\frac{1}{\ln m}$ is the Boltzmann constant or information entropy coefficient, *p*_*ij*_ is the normalized value of the original data of sample point *j* in the second-level index *i* decomposed by the first-level index, *i* = 1, 2, 3, …*n*, *j* = 1, 2, 3, …*m*, and $p_{ij}=k_{ij}/\sum\limits_{i=1}^mk_{ij}$. The positive and negative signs of the formula are set following the principle that internal entropy increases to be positive and external entropy flow to be negative.

### Potential function of the carbon trading system in abrupt change theory

Catastrophe theory was established by René Thom (1972), a French mathematician. It usually refers to the sudden change of the whole system state caused by the continuous change of some variables in the evolution process of the system, which makes the system jump from one stable state to another stable state. Catastrophe theory mainly includes potential, singularity, and attractor. The potential is at its heart. The potential is defined differently in different fields of study. In the field of social sciences, potential represents a certain evolutionary trend of a system. Catastrophe theory is the theory of singularities of potential functions. It studies the abrupt change of the system by the potential function. The potential function of a system can be described by different state variables and control variables. A state variable is the spatial dimension of a trend in a system. The control variable is the spatial dimension of the set of control factors at the time the system runs. Its change determines the trend of the system. That is, the membership function value of the control variable determines the membership function value of the state variable. According to Thom (1972), a maximum of seven mutation forms will occur if the control variables are within four, namely seven primary mutations (Rosen 1972, 1977). After the dissipative characteristics of the system are determined, the risk measurement of the system can be studied with mutation theory.

For the carbon trading market, this paper selects the peak mutation model^4^ to study the risk mutation point and mutation scale. The reason is the dissipative structure of the carbon trading market. The carbon trading market can be understood as a nonlinear complex system that constantly evolves according to the law of entropy increase. The risk time point and size can be set as the state variable *X*. The internal entropy increase and external entropy flow are set as two control variables *u*, *v*. The potential function of the system is as follows (Li 2020; Li 2009; Yu 2021):

4 $$V(x)={x}^4+u{x}^2+ vx$$

Take the first derivative of Eq. (4). Let *V*^′^(*x*) = 0, get the system mutation aggregation shape *M*:

5 $${V}^{\prime }(x)=4{x}^3+2 ux+v=0$$

Calculate the second derivative of Eq. (4). Let *V*^″^(*x*) = 0. Obtain the system singularity set manifold *N*:

6 $${V}^{{\prime\prime} }(x)=12{x}^2+2u=0$$

Combine Eqs. (5) and (6) to obtain the set of bifurcation points^5^. The root discriminant is as follows:

7 $$\varDelta =8{u}^3+27{v}^2$$

The bifurcated set divides the equilibrium surface *M* into two regions. The equilibrium or nonequilibrium position in each region is determined by the sign of discriminant (7). When Δ > 0, the equilibrium surface equation *M* has a real root. The potential function of the system has only one equilibrium position. When Δ < 0, three unequal real roots will appear in the equation of equilibrium surface *M* (Li 2009; Yu 2021; Jin 2019). The potential function of the system has a maximum and two minima. There are three corresponding equilibrium positions. The equilibrium position corresponding to the two minima is the stable state of the system. The equilibrium position corresponding to a maximum value is an unstable state. Under the action of a nonlinear mechanism, the system breaks the original equilibrium state and jumps to another equilibrium state.

### Time-point measurement of risk based on the entropy increase theorem and potential function

According to the analysis in the “Total entropy change of the carbon trading system in dissipation theory” and “Potential function of the carbon trading system in abrupt change theory” sections, the entropy change model is connected with the potential function model. Let *x* be the risk state variable of the carbon sink trading system. *u* = *ds*_*I*_ and *v* = *ds*_*E*_ are the two control variables of the system. The discriminant Δ = 8*u*^3^ + 27*v*^2^ is the mutation criterion of the model. When the control variable *u* > 0, *v* crosses the bifurcation set, then Δ changes from positive to negative or from negative to positive. The overall risk profile of the carbon sink trading system has changed dramatically.

8 $$\left\{\begin{array}{c}u=d{s}_I=\sum {w}_id{s}_I+{c}_i\\ {}v=d{s}_E=\sum {w}_ed{s}_E+{c}_e\end{array}\right.$$

*w*_*i*_ and *w*_*e*_ represent the weight of the corresponding entropy. *c*_*i*_ and *c*_*e*_ represent the corresponding correction constants.

Equation (8) is substituted into Eqs. (4) and (7) to obtain the potential function and sudden change criterion function of the carbon trading system, respectively.

9 $$V(x)={x}^4+\left(\sum {w}_id{s}_I+{c}_i\right){x}^2+\left(\sum {w}_ed{s}_E+{c}_e\right)x$$

10 $$\Delta =8{\left(\sum {w}_id{s}_I+{c}_i\right)}^3+27{\left(\sum {w}_ed{s}_E+{c}_e\right)}^2$$

When Δ < 0, the system mutates. According to Eq. (10), the risk mutation point can be determined.

### Scale measure of risk abrupt change based on information entropy and potential function equilibrium surface equation

The measure of the risk scale is based on information entropy and the potential function equilibrium surface. Information entropy is understood as an index to measure the degree of ordering of a system. The more ordered a system is, the lower the entropy of information. Conversely, the more chaotic a system is, the higher the entropy of information. After 1948, C. E. Shannon further concretized the definition of information entropy (Shannon 2001). Information entropy is the probability of occurrence of discrete random events. It is equal to the product of the entropy value and entropy weight (Jin 2019; Feng et al. 2022; Yang et al. 2021).

Let *R*_*i*_ be the risk scale index of mutation point *i*. It can be expressed numerically as

11 $$R_i=\sum\limits_{i=1}^nw_j\times p_{ij}$$

*w*_*j*_ is the entropy weight of mutation point *j*. It represents the contribution of the indicator to the overall risk. The greater the value is, the greater the contribution to the occurrence of risk. According to the principle of information entropy, there is the following relationship between the entropy weight *w*_*j*_ of index *j* and the information entropy value *e*_*j*_ of index *j*:

12 $$w_j=\frac{1-e_j}{\sum\limits_{j=1}^n\left(1-e_j\right)}$$

*p*_*ij*_ is the standardized risk judgment matrix. Suppose that there are *n* risk factor evaluation indicators. Each index is time series data with time length *m*. Construct a multifactor synthesis matrix. Considering the different measurement units of each risk index, the normalization of each index is further carried out. To construct a dimensionless multifactor synthesis matrix *A*, *A* is a comprehensive evaluation matrix with multiple indexes and multiple time dimensions. It reflects the degree of subordination of each index to each evaluation subset.

13 $$A={\left({p}_{ij}\right)}_{n\times m}=\left[\begin{array}{cccc}{p}_{11}& {p}_{12}& \cdots & {p}_{1m}\\ {}{p}_{21}& {p}_{22}& \cdots & {p}_{2m}\\ {}\vdots & \vdots & \ddots & \vdots \\ {}{p}_{n1}& {p}_{n2}& \cdots & {p}_{nm}\end{array}\right]$$

After calculating the risk scale index value, the value is normalized to facilitate calculation and qualitative analysis. The normalized risk scale index value ${R}_j^{\prime }$ is obtained. It is between [0,1]. The closer to 1, the greater the risk. Based on experience (Yu et al. 2017; Wang et al. 2013; Shan et al. 2014), risk levels are divided as shown in Table 1.

**Table 1:** Risk classification situation

	Risk scale index ${R}_j^{\prime }$	Degree of risk
1	$0\le {R}_j^{\prime }<0.2$	Extremely low risk
2	$0.2\le {R}_j^{\prime }<0.4$	Low risk
3	$0.4\le {R}_j^{\prime }<0.6$	Medium risk
4	$0.6\le {R}_j^{\prime }<0.8$	High risk
5	$0.8\le {R}_j^{\prime}\le 1$	Extremely high risk

## Empirical research

### Variable selection and data processing

#### Variable selection and analysis

As described in the “Total entropy change of the carbon trading system in dissipation theory” section, this paper selects *ds*_*r*_, the price change rate of carbon assets, as the state variable. Macroeconomic policy *ds*_*p*_, economic development level *ds*_*l*_, energy price change *ds*_*e*_, and climate change *ds*_*c*_ were selected as control variables. We set it as four first-level variable indicators. In addition, set secondary indicators. The secondary index of the price change rate of the state variable is set as the CER price yield rate and EUA price yield rate. The secondary indexes of macroeconomic and industrial development include the S&P 500 index, the FTSE 100 index of Britain, the Frankfurt Index of Germany, the CAC40 index of France, and the Stoke 50 index in Europe (Ma and Feng 2022). The secondary indicators of energy factors include ARA triport thermal coal futures, Brent crude oil futures, and NYMEX natural gas settlement price. The secondary indicators of climate factors include temperature, precipitation, and wind as shown in Table 2.

**Table 2:** Indicators of each variable

Level-one variable		Level-two variable	
Price factor	P	CER	P1
		EUA	P2
Macroeconomic and industrial development level factors	I	Germany DAX	I1
		S&P 500	I2
		European Stoke 50	I3
		France CAC40	I4
		FTSE 100	I5
Energy factor	E	Brent crude oil	E1
		European Three Ports ARA	E2
		NYMEX Natural Gas	E3
Climatic factor	C	Average daily temperature index for Europe	C1
		Average daily wind index over Europe	C2
		Average daily precipitation index for Europe	C3

The selection of the above four first-level variable indicators and 13 second-level variable indicators is based on the following considerations.

1. Price factor. The EU carbon market mainly trades EUA, CER, ERU, and EUA futures contracts. Amongst the four products, carbon emission quota EUA and carbon certified emission reduction CER are the carbon trading products with the largest trading volume, trading volume, position, and liquidity in the global carbon market. There is strong dependence and fungibility between the price of CER and the price of EUA. The change in the EUA carbon price directly affects the change in the CER price. In this paper, the first-level index of the price factor is decomposed into CER and EUA two-level indexes to measure the risk occurrence of a complex carbon sink trading system.
2. Macroeconomic and industrial development level. The level of economic development is closely related to the increase or decrease in carbon emissions. During a period of rapid economic development, carbon emissions increase as demand for energy and raw materials increases and output increases. In a recession, carbon emissions fall accordingly. The level of global macroeconomic and industrial development is mainly reflected in the financial market security index. Therefore, the main indexes of the financial markets of the USA and the European Union are selected as the secondary indexes. The Standard & Poor’s 500 index was selected for the U.S. financial market. The reason is that the S&P 500 contains more companies, is more diversified than the Dow, and is weighted by market capitalization rather than the Dow’s share price. In the EU financial market, it selected the FTSE 100 index of Britain, Frankfurt Index of Germany, CAC40 index of France, and Stoke 50 index of Europe. Britain, Germany, and France are important economies in the EU system. Its financial index directly reflects the level of economic development. The reason for choosing the Euro Stoke 50 index is that the fluctuation of the index not only reflects the economic development of the European Union but also affects the economic development of West Asian and North African countries.
3. Energy factor. Fossil fuels include coal, oil, and natural gas. They are the main source of carbon emissions and have a direct and significant impact on the carbon trading market. In this paper, ARA triport thermal coal futures, Brent crude oil futures, and NYMEX natural gas settlement price are selected as the secondary energy price indicators. The reason is that the three ports of Europe thermal coal futures contract is the global thermal coal price benchmark and has a significant global influence. Brent crude oil futures are the benchmark for oil prices in the crude oil market. It is widely traded in futures, over-the-counter swaps, forwards, and spot markets. More than 65% of the world’s physical crude is also priced in the Brent system. The NYMEX natural gas futures contract price is widely used as the benchmark price of natural gas and has an important impact on the global electricity market price.
4. Climatic factor. The climate factor is an important indicator for studying the price fluctuation of carbon assets. In addition to climate policy, natural climate change seriously affects the fluctuation of carbon prices. In this paper, temperature, wind, and precipitation are selected as the secondary indexes of climate factors. High temperature or cold can increase the cooling or heating of power equipment, leading to increased electricity consumption and increased carbon emissions. Wind can affect the amount of renewable energy produced. If the amount of electricity generated from renewable sources falls, the demand for electricity generated from fossil sources will increase, leading to an increase in carbon emissions. Precipitation directly affects the amount of electricity generated from hydropower and increases or decreases the demand for electricity generated from fossil fuels.

In this paper, the daily average temperature index of Europe is constructed by choosing the daily temperature of Britain, Germany, France, Italy, and Spain. The reason is that the climate samples composed of these five countries can represent the climate types of most EU countries. In this paper, the average daily mean temperature index of all meteorological stations in the five countries is selected and summed to obtain the daily mean temperature index of Europe. The European Daily Average Wind Index uses daily average wind data for the UK, Germany, France, Sweden, Turkey, Spain, and Italy. Between 1992 and 2021, these countries accounted for an average of more than 69% of the EU’s total wind power generation. In this paper, the average daily wind power of all meteorological stations in each country is selected to obtain the wind power index of each country, and the weighted sum of wind power generation of each country can obtain the average daily wind power index of Europe. The average daily precipitation in Europe is selected for Austria, France, Norway, Sweden, Switzerland, Turkey, and Italy. Between 1965 and 2021, these countries on average accounted for more than 70% of the EU’s total hydropower generation. The average daily precipitation for all weather stations in each country is averaged. Then, the European average daily precipitation index is obtained by weighted summation with the proportion of hydropower generation in each country.

#### Data sources and processing

The data in this article mainly come from the Bloomberg and Wind databases. The daily closing price data of CER and EUA futures are from the European Climate Exchange. The data on macroeconomic and industrial development levels, as well as energy prices, are sourced from the Oriental Wealth Choice database. Climate data come from the Blended ECA dataset database.

**Fig. 1:**
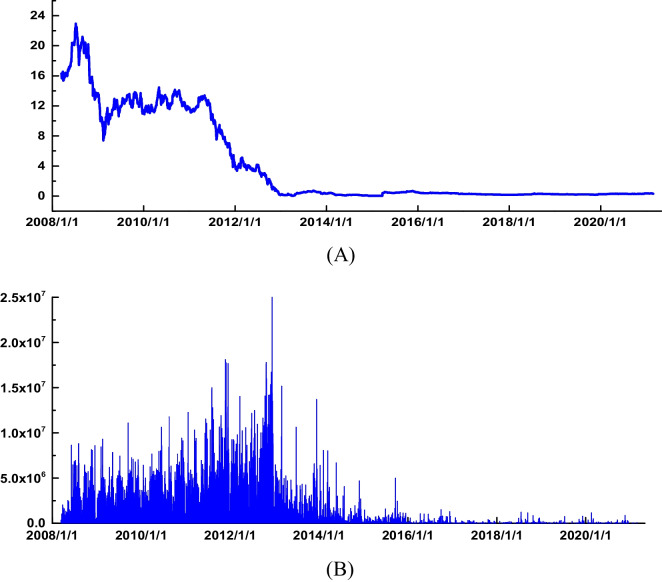
**A** Daily price trends of CER. **B** Daily turnover trend of CER

Since December 2012, CER prices and turnover have been depressed for a long time. The smoothness of the price curve cannot reflect the price fluctuation (Fig. 1(A) and (B)). After 2020, volume and prices fall back. Data from January 1 to March 5, 2021 were excluded. The sample interval is divided into two phases. The first phase is from March 14, 2008 to December 31, 2012. The second phase runs from January 1, 2012 to December 31, 2020. The total number of processed samples was 3361.

To ensure the modelling effect, 13 groups of variable data were integrated based on the daily future prices of CER and EUA. Eight extreme values with yields greater than 0.15 and less than −0.15 were excluded. The average value of five trading days before and after the outlier is used to achieve the smoothing effect. Considering the characteristics of the random cusp mutation model, the winsorize method was used to reduce the tail of variables by 1% and 99% to eliminate the influence of extreme values on the modelling. Thirteen groups of variable data were normalized.

#### Descriptive statistics of the index system

The descriptive statistical results of the index system are shown in Table 3. (1) The yield series has obvious skewness and excess kurtosis (much more than 3). It shows that the yield series has a significant peak and thick tail, and CER has a more obvious peak and thick tail. (2) The J-B statistic is significant at the 1% level, rejecting the original hypothesis that 13 groups of variable sequences obey a normal distribution. (3) The ADF test showed that except for FTSE 100 and ARA in Europe, the other 11 sequences rejected the null hypothesis at the significance level of 5%. That is, time series are stationary series. (4) L-B statistics show that the CER yield time series cannot reject the null hypothesis at the significance level of 5%, and the other 12 groups of variable series all reject the null hypothesis at the significance level of 1%. The results show that there is obvious autocorrelation in 12 groups of variable index sequences. (5) The ARCH-LM statistic shows that the *P* value of the time series of Brent crude oil, European Three Ports ARA, and European daily average temperature index is less than 0.05. The residual has an obvious ARCH effect and conditional heteroscedasticity. The *P* value of the other ten groups of time series was greater than 0.05, which could not reject the null hypothesis and did not have conditional heteroscedasticity.

**Table 3:** Descriptive statistical results of the variable indicators

Variable indicators	Mean	Max	Min	SD	Skewness	Kurtosis	J-B	ADF	L-B	ARCH-LM
CER return rate	2.1	30.0	0.0	3.4	3.1	16.5	30,689.35 (0.00)	−8.593993 (0.00)	409.79 (0.00)	3.774772 (0.0521)
EUA return rate	2.2	35.3	0.0	2.3	3.5	29.6	105,490 (0.00)	−9.156655 (0.00)	220.52 (0.00)	0.314748 (0.5748)
S&P 500	1963.3	3934.8	676.5	750.9	0.4	2.3	181 (0.00)	−3.857436 (0.0139)	3352.9 (0.00)	0.581768 (0.4457)
Germany DAX	9272.6	14,109.5	3666.4	2768.7	−0.1	1.7	249 (0.00)	−3.689312 (0.0231)	3352.7 (0.00)	0.466741 (0.4945)
France CAC40	4390.2	6111.2	2519.3	801.3	0.0	2.0	129 (0.00)	−3.871844 (0.0133)	3342.2 (0.00)	1.031246 (0.3099)
FTSE 100	6272.7	7877.5	3512.1	900.1	−0.5	2.9	167 (0.00)	−3.148785 (0.0952)	3343.5 (0.00)	1.442211 (0.2285)
European Stoke 50	3044.8	3882.3	1810.0	434.7	−0.3	2.3	130 (0.00)	−3.862975 (0.0137)	3330.2 (0.00)	1.275793 (0.2588)
Brent crude oil	1.6	24.4	0.0	1.8	3.6	28.7	99,743 (0.00)	−7.065222 (0.00)	329.53 (0.00)	9.096894 (0.0026)
European Three Ports ARA	78.2	131.1	38.0	20.9	0.4	2.7	108 (0.00)	−2.196977 (0.4906)	3084.5 (0.00)	5.678914 (0.0172)
NYMEX Natural Gas	2.2	53.0	0.0	2.2	5.9	98.3	1,291,055 (0.00)	−10.08256 (0.00)	36.019 (0.00)	1.046694 (0.3063)
Daily average temperature in Europe	12.1	26.4	−4.4	6.2	0.0	2.0	153 (0.00)	−4.036123 (0.0078)	3233.8 (0.00)	2.842699 (0.0364)
Daily average wind speed in Europe	3.5	7.4	2.1	0.7	1.1	4.8	1069 (0.00)	−22.52343 (0.00)	1332.2 (0.00)	0.249504 (0.7792)
Average daily precipitation in Europe	2.6	14.0	0.0	1.8	1.4	6.0	2465 (0.00)	−39.51225 (0.00)	448.17 (0.00)	1.220414 (0.2952)

### Detection and analysis of risk time points

#### Detection of risk time points

**Fig. 2:**
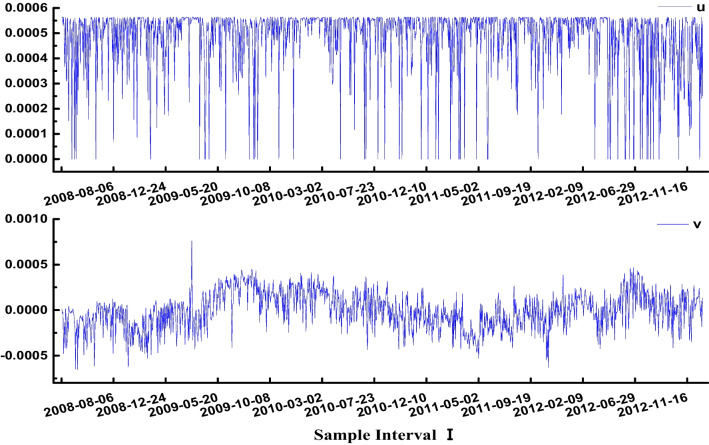
Control variables *u* and *v* of sample interval I

**Fig. 3:**
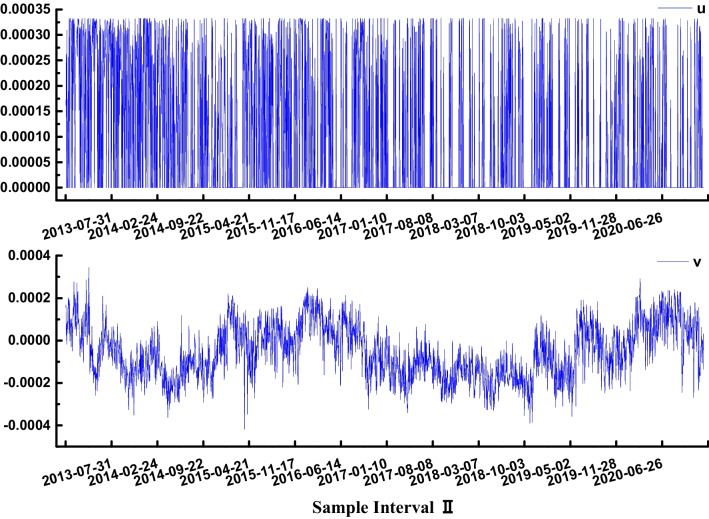
Control variables *u* and *v* of sample interval II

Substitute the data from sample interval I and sample interval II into Eq. (8) to obtain the control variable *u* = *ds*_*i*_ (internal entropy increase) and control variable *v* = *ds*_*e*_ (external entropy flow) of the carbon sink trading system. The results are shown in Figs. 2 and 3.

From Fig. 2, in the sample interval stage I, the internal entropy increase of the carbon sink trading market fluctuates within the range of 0.0000 to 0.00058, *ds*_*i*_ > 0. The external entropy flow fluctuates in the range of −0.0005 to 0.00057, *ds*_*e*_ ≠ 0. This shows that the system has typical dissipative structural characteristics. Its internal entropy increase has a decisive effect on the disordered operation of the whole system. The influence of external entropy flow on the disorderly motion of the system is less than that of internal entropy increase. It is consistent with the characteristics of sample interval I with active trading, large trade volume and holdings, and abundant price upwards momentum.

From Fig. 3, in sample interval II, the internal entropy increase fluctuates frequently in the relatively small range of 0.000 to 0.00032, *ds*_*i*_ > 0. The external entropy flow fluctuates in the range of −0.0004 to 0.00035, *ds*_*e*_ ≠ 0. It reflects the dissipative structure of the system. However, compared with sample interval I, the price fluctuation interval is obviously narrow. This indicates that the influence of both internal entropy increases and external entropy flow on the disorderly operation of the whole system is smaller than that of sample interval I. This is mainly due to the international carbon emission reduction policies in the post-Kyoto era, the change in the EU carbon trading mechanism, energy, local wars, and other factors that lead to the shrinkage of the global carbon trading market and long-term low carbon trading prices. It is consistent with declining volumes and positions. It shows that the total entropy change model can effectively capture the motion development track of the carbon sink trading system.

**Fig. 4:**
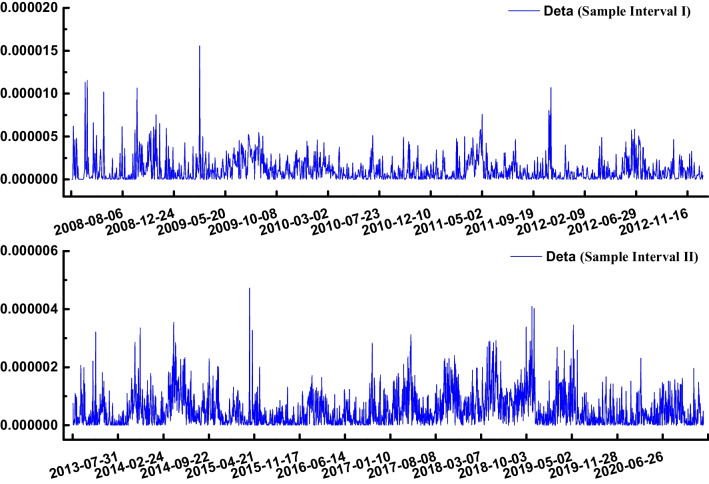
Discriminant ∆ changes of sample intervals I and II

The above two control variables are substituted into the discriminant Eq. (10) of the entropy mutation model. The mutation result is shown in Fig. 4 (the discriminant ∆ is represented by delta).

Figure 4 shows that when the control variable satisfies discriminant *Δ* = 8(∑*w*_*i*_*ds*_*I*_ + *c*_*i*_)^3^ + 27(∑*w*_*e*_*ds*_*E*_ + *c*_*e*_)^2^ < 0, the state variable is located in the nonstationary region of the equilibrium surface. First, the position of Δ < 0 is obtained to determine whether risk mutations occur. According to mutation theory, when the control variable *u* > 0, the control variable *v* will cross the bifurcation set and change from a positive value to a negative value and then to a positive value, and the system will have the mutation. It can be seen that there are a total of 36 points with Δ < 0 in sample interval I. According to the jump and delay convention characteristics, the equilibrium surface equation *M* has three different real roots when Δ < 0. The potential function of the system has one maximum and two minima. The equilibrium position system corresponding to the two minima is in a stable state. The equilibrium position corresponding to a maximum is unstable. There are 12 risk mutation points as shown in Table 4.

**Table 4:** The mutation points of sample range I

	Time		Time
1	2008-05-29	7	2009-02-06
2	2008-07-08	8	2009-09-30
3	2008-07-24	9	2010-05-17
4	2008-07-28	10	2010-07-12
5	2008-08-13	11	2011-12-09
6	2008-09-01	12	2012-12-21

Similarly, there are 12 points with Δ < 0 in sample interval II. There are four risk mutation points as shown in Table 5.

**Table 5:** The mutation points of sample range II

	Time		Time
1	2013-08-07	3	2015-06-25
2	2015-04-29	4	2016-05-24

#### Analysis of inducing factors of risk mutation points

The abrupt change in risk in the EU carbon trading market was mainly influenced by the global financial crisis in 2008, the European sovereign debt crisis in 2010, and the new EU energy efficiency plan in 2011.

There are a total of 12 price risk mutation points in sample interval I. The first to eighth risk mutation points occurred during the financial crisis period from May 2008 to September 2009. This is a global financial crisis triggered by the US subprime mortgage crisis. In mid-January 2008, Citigroup, Merrill Lynch, JPMorgan Chase, and Swiss Bank suffered severe losses. In February, the G7 finance ministers and central bank governors meeting statement noted that the impact of the subprime crisis has increased. On March 27, 2008, the European money market liquidity crisis again, the Bank of England and the Swiss National Bank jointly injected capital. On April 8, 2008, the IMF reported $1 trillion in global subprime losses. The OECD forecasts global subprime losses of $350–420 billion. In August, the share prices of Fannie Mae and Freddie Mac plunged, and the financial institutions that held their debt suffered massive losses. On September 15, 2008, Lehman Brothers, America’s fourth-largest investment bank, filed for bankruptcy protection. On the same day, the three major international rating companies collectively downgraded AIG’s credit rating. AIG’s stock price plummeted by 60%. The US financial crisis erupted globally. In October, Iceland suffered a major banking and financial crisis. The exchange rate of the Icelandic krona against the euro depreciated by 30%. The country is bankrupt. Britain, Germany, Japan, South Korea, and other countries have suffered heavy losses in the stock market and import and export declines. The currencies of some emerging markets and other developing countries have depreciated sharply. On October 8, the European Central Bank, the Bank of England, the Federal Reserve, the Bank of Canada, the Bank of Sweden, and the Swiss Bank all cut their benchmark interest rates by 0.5%. In November, the Chinese government introduced a series of fiscal and monetary policies. A special fund of 4 trillion yuan was provided to cope with the 2008 economic crisis. Through the efforts of the Federal Reserve and governments around the world, the financial crisis was finally declared over in March 2010. During this period, carbon assets, as a kind of financial asset, are inevitably affected by the financial crisis. In particular, the bankruptcy of carbon trading-active financial companies such as Lehman Brothers and Bear Stearns has led to a sharp decline in carbon market trading volume and demand. Market investors rushed to sell their carbon allowances, resulting in a huge surplus of allowances, and prices plummeted. At the same time, many enterprises went bankrupt during the financial crisis. The output of energy-intensive industries declines, carbon emissions decrease, and oil and other energy prices continue to fall, eventually leading to lower demand for carbon assets and insufficient liquidity in the market. Although there was a brief recovery in EU ETS trading volumes in early 2009, the plunge in carbon prices could not be contained.

The ninth to twelfth risk mutation points were during the European debt crisis. The crisis began with Greece’s sovereign debt crisis. In October 2009, the Greek government announced that its fiscal deficit and public debt would reach 12.7% and 113% of GDP, respectively. This far exceeds the upper limits of 3% and 60% set by the EU’s Stability and Growth Pact. It has triggered price fluctuations in other financial market assets. Standard & Poor’s, Moody’s Investors, and Fitch downgraded Greece’s long-term sovereign credit rating in December 2009. As a result, the Greek government debt crisis has sparked international concerns that the debt crisis will spread to all of Europe and then affect the global economy, which is gradually recovering from the financial crisis. In December 2009, Greece sold 2 billion euros of bonds. Then, the rest of Europe began to fall into crisis. Spain, one of the euro zone’s stronger economies, also expects its budget deficit to rise over the next 3 years. In February 2010, the debt crisis triggered a stock market crash in Spain. Since then, the euro has fallen sharply, European financial assets have plummeted, and Germany and other eurozone countries have been affected by the crisis. Finance ministers from the 27-nation European Union were forced to approve a 750 billion-euro rescue package to help eurozone members at risk of falling into debt crisis and prevent the crisis from spreading. In November 2010, the European Union approved an 85 billion-euro rescue package for the Irish government. Portugal received a 78 billion-euro bailout package from the European Union in May 2011. At the same time, the Greek debt crisis has created new problems in paying maturing public debt, which has triggered a series of crises. The crisis was brought under control only after the European Union promised an aid package. In 2012, European countries hit a debt servicing peak. The European debt crisis is spreading further. Portugal, Ireland, Italy, Greece, and Spain have maturing debt of 564.2 billion euros. In January, the 25 countries of the European Union signed a fiscal compact. Standard & Poor’s has downgraded the long-term credit ratings of nine eurozone countries. In February, Greece reached a second bailout loan agreement with the European Union and the International Monetary Fund. In June, Germany, France, Italy, and Spain agreed to a 130 billion-euro stimulus package. In July, the Eurogroup approved a rescue plan for Spain’s banks. In October, the European Stability Mechanism came into force. The debt repayment crisis has been exacerbated by excessive international borrowing by some European countries since the 2008 financial crisis. The whole European economy has suffered. Carbon financial assets languished at a low level during this period. By the end of 2012, the price of a CER had fallen to 0.5 euros.

There are two reasons for the four risk mutation points in sample interval II mainly: (1) the new energy efficiency plan issued by the European Union. In March 2011, the European Union issued a new energy efficiency plan. From a supply perspective, the new energy efficiency program has led to a large increase in international emission reduction projects, resulting in an oversupply of CERs. At the same time, the program mandates that enterprises adopt energy-saving and emission-reduction measures, and enterprises operate less, reducing carbon emissions. The demand for CER in the carbon market decreases, and the price of carbon assets is expected to decline and hover at a low level for a long time. The trading is abnormally low, and the position and trading volume drop significantly. In addition, the European Union imposes strict controls and restrictions on the amount of CER offset and the types of CER projects, which further reduces the overall market demand. Eventually, the market for CER in the European Union’s emission trading system collapsed and almost stopped trading. (2) The international carbon emission reduction policy in the post-Kyoto era, the change of the EU carbon trading mechanism, and the influence of various factors such as energy and local wars have led to the contraction of the global carbon trading market and the long-term downturn in carbon trading prices, which is consistent with the actual situation of declining volume and position. It shows that the total entropy change model can capture the trajectory of the carbon sink trading system.

### Measurement of risk scale

The assessment steps for the risk scale are as follows.

The first step is to establish and standardize the risk assessment matrix. In the second step, information entropy is calculated according to Eq. (3). The third step is to calculate the entropy weight of the risk evaluation index according to Eq. (12). The fourth step is to calculate the risk scale index according to Eq. (11). The fifth step is to determine the risk level and evaluate the risk scale.

#### Measurement of risk scale at each mutation point

According to Eqs. (3) and (12), the entropy value *Q* and entropy weight *W* of each mutation point are determined as shown in Tables 6 and 7.

**Table 6:** Entropy and entropy weight of the variable index (sample range I)

Level-one variable	Level-two variable	Entropy (*e*)	Entropy weight (*w*)
Price factor	CER	0.2169	0.5052
	EUA	0.2330	0.4948
Macroeconomic and industrial development level	Germany DAX	0.2521	0.2063
	S&P 500	0.2362	0.2107
	European Stoke 50	0.3215	0.1872
	France CAC40	0.3226	0.1869
	FTSE 100	0.2426	0.2089
Energy factor	NYMEX Natural Gas	0.1724	0.3426
	European Three Ports ARA	0.2057	0.3288
	Brent crude oil	0.2060	0.3286
Climatic factor	Average daily temperature index for Europe	0.2806	0.3128
	Average daily wind index over Europe	0.2146	0.3415
	Average daily precipitation index for Europe	0.2051	0.3457

**Table 7:** Entropy and entropy weight of the variable index (sample range II)

Level-one variable	Level-two variable	Entropy (*e*)	Entropy weight (*w*)
Price	CER	0.0918	0.5392
	EUA	0.2240	0.4608
Macroeconomic and industrial development level	Germany DAX	0.2565	0.2056
	S&P 500	0.2967	0.1945
	European Stoke 50	0.2775	0.1998
	France CAC40	0.2917	0.1959
	FTSE 100	0.2618	0.2042
Energy	NYMEX Natural Gas	0.1106	0.3606
	European Three Ports ARA	0.2798	0.2920
	Brent crude oil	0.1434	0.3474
Climatic	Average daily temperature index for Europe	0.2711	0.3089
	Average daily wind index over Europe	0.2120	0.3339
	Average daily precipitation index for Europe	0.1568	0.3573

The risk scale index and risk grade corresponding to the abrupt change time point are shown in Tables 8 and 9.

**Table 8:** Risk value and risk grade at the point of mutation (sample range I)

	Time	Risk scale index (*R*_*j*_)	Normalized risk scale index (${R}_j^{\prime }$)	Risk level
1	2008-05-29	1.6477	0.7378	High risk
2	2008-07-08	1.8932	0.9746	Extremely high risk
3	2008-07-24	1.5720	0.6647	High risk
4	2008-07-28	1.6097	0.7011	High risk
5	2008-08-13	1.2810	0.3840	Low risk
6	2008-09-01	1.5574	0.6507	High risk
7	2009-02-06	0.8830	0.0000	Extremely low risk
8	2009-09-30	1.4738	0.5700	Medium risk
9	2010-05-17	1.5681	0.6610	High risk
10	2010-07-12	1.5839	0.6762	High risk
11	2011-12-09	1.3344	0.4355	Medium risk
12	2012-12-21	1.9195	1.0000	Extremely high risk

**Table 9:** Risk value and risk grade at the point of mutation (sample range II)

	Time	Risk scale index (*R*_*j*_)	Normalized risk scale index (${R}_j^{\prime }$)	Risk level
1	2013-08-07	1.1580	0.1086	Low risk
2	2015-04-29	1.3751	0.6658	High risk
3	2015-06-25	1.5053	1.0000	Extremely high risk
4	2016-05-24	1.1157	0.0000	Extremely low risk

#### Risk scale analysis

Tables 8 and 9 show that amongst the 12 risk mutation points in sample interval I, except for one extremely low risk point, one low-risk point, and two moderate-risk points, the remaining eight mutation points are all high risk or above. There are two extremely high risk points. This indicates that the model effectively captures the time points at which the risks of the financial crisis and the European debt crisis occur. Amongst the four risk scale measures in sample interval II, there are two risk points with high risk or above. There is one low-risk point and one extremely low risk point each. The scale of high risk is mainly caused by the following factors: (1) the collapse of the Chinese stock market. In June 2015, China’s stock market experienced a huge crash, with the Shanghai Composite index plunging nearly 30 percent in 2 weeks. Despite a series of measures taken by the Chinese government to stabilize the market, it still triggered a global market panic. (2) Greek debt crisis. Greece continued to face a debt crisis in 2015. In July 2015, the Greek government announced that it would not be able to repay its massive debt, an event that triggered tensions between Greece and its international creditors and eventually led to two emergency elections in Greece in July and August 2015. (3) The US Federal Reserve System (Fed) to raise interest rates. In 2015, the Fed announced the end of the zero interest rates policy for several years and began to gradually raise interest rates. This move marked the recovery of the US economy and had a huge impact on the global market, causing concern and volatility in the global market. (4) Global commodity price declined. In 2015, global commodity prices such as crude oil, metals, and food generally fell, which was mainly caused by the global economic contraction, oversupply, and China’s economic slowdown, which had a serious impact on the economies and markets of many developing countries. (5) Economic slowdown in emerging markets. Growth slowed in some emerging markets in 2015, notably Brazil and Russia.

In summary, there are 16 risk mutation points between 2008 and 2021. Amongst them, 62.5% were at high risk or above. It shows that the financial crisis and the European debt crisis directly led to the carbon market price jump.

## Research conclusion and enlightenment

1. According to the theory of total entropy change of dissipative structure, this paper defines the carbon trading market as a nonlinear complex system that constantly evolves following the law of entropy increase. This paper constructs an index system with carbon price yield as internal entropy increase and macroeconomic policy and economic development level, energy price, and climate change as external entropy flow. The system has 4 primary indexes and 13 secondary indexes. At the same time, combined with the potential function of catastrophe theory, the risk time point and scale are set as state variables. The internal entropy increase and external entropy flow are set as two control variables. This paper studies the time point and scale of risk in the carbon trading market and makes an innovative discussion in theory.
2. Based on the entropy of dissipation theory and the potential function of sudden change theory, the risk sudden change point measurement model is constructed to effectively detect 16 risk sudden change points in the financial crisis, the European debt crisis, and the European new energy efficiency plan. The empirical test shows that the model has a good ability to capture mutation points and prediction accuracy, and the fitting effect is good. The results support the accuracy and science of the technique.
3. Risk scale measurement technology based on information entropy and the potential function surface equation can effectively calculate the risk index value of the risk mutation point and then judge the degree and grade of risk. Between 2008 and 2021, there were three extremely high risks, seven high risks, two moderate risks, two low risks, and two very low risks, with 62.5% of the total being high risk or above. This shows that the impact of an external shock on the market price cannot be underestimated.

## Data Availability

Raw data can be downloaded from Bloomberg database (https://www.bloomberg.org/) and Wind database (https://www.wind.com.cn/). Details of all data and procedure used in the analysis are available in the main text or on request of the corresponding authors. No restrictions are placed on materials.

## Footnotes

1 BlueNext was once the largest spot market for carbon dioxide emission rights, headquartered in Paris, France. On October 26, 2012, BlueNext announced that it would permanently close its carbon spot and carbon derivative trading business on December 5, 2012.

2 In 1865, German physicist R. J. E. Clausius proposed the concept of entropy. It was used to measure the problem of system energy degradation. In the field of dynamics, when the overall entropy of a system increases, its function will relatively decrease.

3 In 1948, American mathematician C.E. Shannon introduced the concept of entropy into information theory and defined it as information entropy. Information entropy is a kind of entropy of system uncertainty degree expressed by probability. The greater the system uncertainty, the greater the information entropy. The more disordered the system is.

4 The peak mutation model is a potential function model with one state variable and two control variables. It is consistent with the entropy increase theorem variable of carbon trading market risk state modelling.

5 The bifurcation set is the value corresponding to the control variable when the mutation occurs.

## Declarations

### Disclaimer

The writers are responsible for the consequences of this article.
